# Antioxidant and Protective Effects of the Polyphenolic Glycoconjugate from *Agrimonia eupatoria* L. Herb in the Prevention of Inflammation in Human Cells

**DOI:** 10.3390/jfb14040182

**Published:** 2023-03-26

**Authors:** Marta Tsirigotis-Maniecka, Ewa Zaczyńska, Anna Czarny, Piotr Jadczyk, Barbara Umińska-Wasiluk, Roman Gancarz, Izabela Pawlaczyk-Graja

**Affiliations:** 1 Department of Engineering and Technology of Chemical Processes, Wrocław University of Science and Technology, Wybrzeże Wyspiańskiego 29, 50-370 Wrocław, Poland; 2 Department of Experimental Therapy, Hirszfeld Institute of Immunology and Experimental Therapy, Polish Academy of Sciences, Laboratory of Immunobiology, R. Weigla Str. 12, 53-114 Wrocław, Poland; 3 Department of Environmental Protection Engineering, Wrocław University of Science and Technology, Wybrzeże Wyspiańskiego 27, 50-370 Wrocław, Poland

**Keywords:** polyphenolic-polysaccharide complex, complex biopolymer, antioxidant, noncytotoxic, noninflammatory, nongenotoxic, nonmutagenic

## Abstract

Herein, structural and biological studies of a complex biopolymer (polyphenolic glycoconjugate) isolated from the flowering parts of *Agrimonia eupatoria* L. (**AE**) are presented. Spectroscopic analyses (UV–Vis and ^1^H NMR) of the aglycone component of **AE** confirmed that it consists mainly of aromatic and aliphatic structures characteristic of polyphenols. **AE** showed significant free radical elimination activity, i.e., ABTS^+^ and DPPH^·^, and was an effective copper reducing agent in the CUPRAC test, eventually proving that **AE** is a powerful antioxidant. **AE** was nontoxic to human lung adenocarcinoma cells (A549) and mouse fibroblasts (L929) and was nongenotoxic to *S. typhimurium* bacterial strains TA98 and TA100. Moreover, **AE** did not induce the release of proinflammatory cytokines such as interleukin 6 (IL-6) and tumor necrosis factor (TNF-α) by human pulmonary vein (HPVE-26) endothelial cells or human peripheral blood mononuclear cells (PBMCs). These findings correlated with the low activation of the transcription factor NF-κB in these cells, which plays an important role in the regulation of the expression of genes responsible for inflammatory mediator synthesis. The **AE** properties described here suggest that it may be useful for protecting cells from the adverse consequences of oxidative stress and could be valuable as a biomaterial for surface functionalization.

## 1. Introduction

Polyphenols are well known for their many valuable biological activities, such as free radical scavenging, and their antioxidant activity is considered to be one of their main health-promoting properties. Therefore, polyphenols are believed to protect living organisms against oxidative stress by reducing the activity of reactive oxygen species (ROS) [1]. The disturbance of the balance between the production and destruction of ROS results in an uncontrolled increase in its concentration. This can lead to oxidative stress, which is responsible for direct damage to important biomacromolecules, i.e., lipids, nucleic acids, and proteins [2,3]. Prolonged oxidative stress contributes to the emergence of many diseases in society, including cardiovascular complications and neoplastic disorders [4,5]. Even moderate oxidative stress cause platelet activation, resulting in uncontrolled blood clot formation as well as the release of various inflammatory mediators, including proinflammatory cytokines such as interleukin 6 (IL-6) and tumor necrosis factor α (TNF-α) [6]. Therefore, daily intake of polyphenols indirectly but very effectively reduces the risk of many health problems [7]. The properties of polyphenolic compounds that are valuable for human health include the ability of their hydroxyl groups to form hydrogen bonds with many other molecules, e.g., with the protons of the aromatic rings present in the structures of proteins, which reduces the activity of enzymes involved in ROS generation such as cytochrome P450, cyclooxygenase, lipoxygenase, or xanthine oxidase [8,9,10,11]. Moreover, polyphenols have the ability to chelate and remove the metal ions that are responsible for the formation of free radicals [8,12]. Among the other well-identified properties of polyphenols, their anti-inflammatory activity [12] results mainly from their capability to inhibit the biosynthesis of factors that cause inflammation and platelet activation, which results in platelet aggregation [13]. Specifically, these factors are prostaglandins, prostacyclins [14], superoxide anions [15], nitric oxide [9], leukotrienes, and isoprostanoids [14].

Scientific reports have indicated that plant products obtained from the aerial parts of *Agrimonia eupatoria* L. have great pharmaceutical and biological potential, including the recently revealed in vitro anticoagulant activity [16], due to the conjugates of pectin-like polysaccharides with macromolecular polyphenolic matrices. Other well-described small molecule polyphenols from *A. eupatoria*, including flavonoids, phenolic acids, coumarins, tannins, and terpenoids, have demonstrated beneficial health effects mostly due to their well-known free radical scavenging properties [13,17,18,19,20,21]. Literature studies have shown that *A. eupatoria* tissues are especially rich in various flavonoid glycosides, such as luteolin, acacetin, apigenin, quercetin, kaempferol, kaempferide, and rutin [17,22,23,24], in addition to some flavonols, i.e., catechin, epicatechin and its polymers, myricetin and isorhamnetin, and phenolic acids, i.e., *p*-coumaric, vanillic, gentisic, *p*-hydroxybenzoic, and hydroxycinnamic acids [16,17,25,26]. Regardless of the expected benefits of many plant polyphenols, some may be cytotoxic [27,28], and as a result, may disturb the functioning of internal organs, including the liver, kidneys, heart, and respiratory system, and even damage the DNA structure [29]. Therefore, it is essential to verify that a plant compound with beneficial properties is safe for internal use.

In light of the current state of knowledge on polyphenols [30], especially those from *A. eupatoria* [16], and their interesting biological properties, we decided to investigate the cytokine modulation effect and in vitro antimutagenic and antioxidant activities of polyphenolic glycoconjugates isolated from the flowering parts of this medicinal plant. The presented research is a continuation of previous studies on the properties of the complex biopolymer of *A. eupatoria* (**AE**). Therefore, we focused on the activities that could arise from the polyphenolic component of the conjugate. We characterized the ability of the polyphenolic–polysaccharide complex to eliminate free radicals by the both hydrogen atom transfer (HAT) and single-electron transfer (SET), as the mechanism of action of plant-derived antioxidants is not fully understood. Furthermore, the activity of the **AE** product in cell lines and bacterial cell strains was examined to assess its possible cytotoxic and mutagenic effects. Moreover, a series of in vitro experiments evaluated the role of **AE** as a stimulant of the nuclear factor κB (NF-κB) system in peripheral blood mononuclear cells (PBMCs) and human pulmonary vein endothelial cells.

## 2. Materials and Methods

### 2.1. Plant Material and Reagents

The flowering parts of *A. eupatoria* L. were purchased from the local market in Poland. The plant identity was certified by Prof. Krystyna Kromer of Wrocław University (Wrocław, Poland), and an herbarium voucher specimen (No. 005054) was deposited in the Wrocław University Botanical Garden (Wrocław, Poland). RPMI-1640, Hank’s, and F12 media were purchased from Biowest (Nuaillé, France). Foetal calf serum (FCS) was obtained from HyClone (Pittsburgh, PA, USA). The BD OptEIA test kit was purchased from BD Biosciences Pharmingen (San Jose, CA, USA). SMF 1077 lymphocyte liquid (1.077 g/mL) was obtained from CytoGen (Łódź, Poland). The primary monoclonal antibody anti-NF-κB p-65 was purchased from Chemicon International Inc. (Billerica, MA, USA). Biotinylated secondary antibody, hydroxide peroxide (30%), and a liquid diaminobenzidine (DAB) substrate kit for peroxidase were purchased from Novocastra Laboratories Ltd. (Newcastle upon Tyne, UK). The 3,3′,5,5′-tetramethylbenzidine (TMB) reagent and trypan blue (0.4%) were purchased from Serva Electrophoresis GmbH (Heidelberg, Germany). Phosphate-buffered saline (PBS) was prepared at the Institute of Immunology and Experimental Therapy (Wroclaw, Poland). Haematoxylin, Novostain Super ABC Reagent, and Ames MPF 98/100 test were purchased from Endotell AG (Alschwill, Switzerland). Additionally, L-glutamine, penicillin and streptomycin solutions, sodium pyruvate, 2-mercaptoethanol, cyclolino peptide A (CLA), lipopolysaccharide (LPS) from *E. coli* (0111:B4, L-4130), endothelial cell growth supplement (ECGS), sodium hydroxide-reduced nicotinamide adenine dinucleotide (NADH), 2,2-azinobis(3-ethylbenzothiazoline)-6-sulfonic acid (ABTS), 2,2-diphenyl-1-picrylhydrazyl (DPPH), neocuproine, 6-hydroxy-2,5,7,8-tetramethylchroman-2-carboxylic acid (Trolox, TR), paraformaldehyde, dimethyl sulfoxide (DMSO), and Tris buffer were purchased from Sigma–Aldrich (Poznań, Poland). Tetramethylammonium hydroxide (TMAH), inorganic salts, and organic solvents of analytical grade purity were obtained from Avantor Performance Materials (Gliwice, Poland). The DAB substrate was prepared using the liquid DAB substrate kit for peroxidase as follows: 4 drops of 0.1% DAB liquid were dissolved in 0.1 M Tris buffer and mixed with 2 drops of 0.02% H_2_O_2_; thereafter, the mixture was diluted in 5 mL of distilled water.

### 2.2. Cell Lines

The human lung adenocarcinoma cell line A549 (ATCC CCL 185) and mouse fibroblast cell line L929 (American Type Culture Collection Certified Cell Line ATCC CCL1) were derived from the Institute of Immunology and Experimental Therapy collection of cell lines (Wroclaw, Poland). L929 and A549 cells were cultured in RPMI-1640 medium with 100 U/mL penicillin, 100 µg/mL streptomycin, 2 mM L-glutamine, and 10% FCS. Human pulmonary vein endothelial cells (HPVE-26, ATCC CRL-2607) were obtained from the American Type Culture Collection (Manassas, VA, USA). HPVE-26 cells were cultured in F12 medium with 100 U/mL penicillin, 100 µg/mL streptomycin, 2 mM L-glutamine, 10% FCS, and 0.03 mg/mL ECGS (Sigma–Aldrich, Saint Louis, MO, USA).

### 2.3. Complex Biopolymer Isolation and Characterization

The process of complex biopolymer isolation from the herb *A. eupatoria* L. (**AE**, M_w_~55 × 10^3^ g/mol) was carried out following a multistep procedure previously described [16]. Briefly, the dried, ground parts of the plant material were minced and extracted twice with hexane for degreasing (4 h, 69 °C). Thereafter, the plant material was macerated in 0.1 M NaOH (24 h, room temperature (RT)) and then extracted with 0.1 M NaOH (4 h, 97 °C). Subsequently, the collected extract was filtrated and centrifuged (15 min, RT, 1850× *g*) and neutralized, and the solvent was evaporated. The obtained extract was dissolved in distilled water and extracted twice with diethyl ether (4 h, 34 °C). Thereafter, the organic fraction was removed, and the aqueous extract was concentrated. The paste-like extract was suspended in methanol (24 h, RT), and the alcohol-insoluble residue (AIR) was filtered off and then dried. The obtained dry product was dissolved in distilled water and dialyzed (MWCO 12.5–14.0 × 10^3^ Da) against distilled water (96 h, RT). Finally, the macromolecular fraction was obtained and evaporated under reduced pressure to give the polyphenolic glycoconjugate (**AE**).

Prior to ^1^H NMR analysis, **AE** was subjected to acidic hydrolysis (trifluoracetic acid (TFA), 120 °C, 5 h) to obtain the aglycone part (**AEag**). The ^1^H NMR spectrum of **AEag** was recorded in 10% NaOD at RT on a Bruker AMX600 NMR spectrophotometer (Rheinstetten, Germany). The chemical shifts were referenced to TMS as an internal standard. The UV–Vis spectrum of **AEag** was recorded in an alkaline solution (C = 0.05 mg/mL) in the wavelength range of λ = 200–1000 nm using a SPECTROstar Nano microplate reader (BMG Labtech, Ortenberg, Germany).

### 2.4. Antioxidant Assays

Three different spectrophotometric tests were used to assess the total antioxidant capacity of **AE**: ABTS [31], DPPH [32], and CUPRAC [33] assays. To estimate the ability of AE to reduce DPPH^●^ oxidant activity, the plant product was dissolved in an aqueous ethanol solution (*v/v*, 1:1) to obtain a series of solutions with concentrations between 2.5 and 1000 µg/mL. Additionally, the antioxidant activity of **AE** (ABTS^+^· assay) and the ability of AE to reduce the Cu(II) ions in neocuproine (CUPRAC test) were assessed by applying 0.5–500 µg/mL aqueous solutions of **AE**. Because the antioxidant activity of **AE** was presumed to be in a range similar to that of plant preparations, TR was used as a positive control. The relative antioxidant activity of **AE** was expressed as a total equivalent antioxidant capacity (TEAC) or as the concentration required to show 50% antioxidant activity, IC^A^_50_ and IC^D^_50_, respectively [34].

### 2.5. Determination of AE Toxicity

#### 2.5.1. Cell line Propagation

**AE** was dissolved in RPMI-1640 medium supplemented with 2% FCS in a range of concentrations (1.9–1000 µg/mL). **AE** cytotoxicity was determined by measuring the growth of the human lung adenocarcinoma cell line A549 and mouse fibroblast cell line L929. The criteria to determine the toxicity effect were based on changes in cell morphology according to EN ISO10993-5:2009 (“Biological Evaluation. Part 5: Test of in Vitro Cytotoxicity, International Organization for Standardization” 2009) [35]. Evaluation of the potential cytotoxicity of **AE** was performed in monolayer cultures of A549 or L929 cells. Each time, cells at a density of 5 × 10^4^/well were incubated for 24 h in a cell culture incubator. Thereafter, the culture supernatant was removed, and an appropriate amount of **AE** sample in culture medium was added to the monolayer cell cultures (200 µL/well) and then incubated at 37 °C in 5% CO_2_ air for 72 h. Cell growth, morphology, and viability (trypan blue staining) were determined using image analysis methods. The degree of cytotoxicity was defined as the highest concentration of **AE** that caused at least 50% cell destruction.

#### 2.5.2. Clinical Evaluation of Patients

The study was approved by the Ethics Committee (Provincial Specialist Hospital in Wrocław, Poland) (approval no KB/nr6/rok2012) and was carried out according to the Declaration of Helsinki. Informed consent was obtained from all subjects/volunteers involved in the study. Complete peripheral blood was collected from volunteers using heparinized syringe systems at the Blood Donation Station of the IV Military Clinical Hospital with Policlinics in Wrocław (Poland). The donors were healthy men aged 19 to 23 years who were diagnosed as free of viral and bacterial infections, inflammation, and allergic diseases, and were not treated with any antiplatelet, anticoagulant, or anti-inflammatory drugs or antibiotics within 14 days prior to blood donation.

#### 2.5.3. Isolation of PBMCs

Venous blood from a single donor was withdrawn into heparinized syringes and diluted twice with PBS. PBMCs were isolated by centrifugation on an SMF 1077 Lymphocyte at 400× *g* for 20 min at 4 °C. The interphase cells were then washed three times with Hank’s medium and resuspended in culture medium at a density of 2 × 10^6^ cells/mL. Isolated cells were cultured in a RPMI-1640 medium supplemented with 10% FCS, antibiotics, 1 mM sodium pyruvate, and 2-mercaptoethanol, and incubated in a cell culture incubator at 37 °C, 5% CO_2_.

#### 2.5.4. Activation of NF-κB in PBMCs

PBMCs were distributed in 96-well flat-bottom plates in 100 µL aliquots (2 × 10^5^ cells/well). LPS from *E. coli* (O111:B4) was added at a concentration of 5 µg/mL. AE was tested at the following concentrations: 10, 50, and 100 µg/mL. The level of NF-κB activation in cells was determined by immunocytochemical staining.

#### 2.5.5. Induction of Cytokines in Human Whole Blood

Human whole blood was diluted 10-fold with RPMI-1640 medium and distributed to 24-well culture plates in 1 mL aliquots. Cultures were stimulated with LPS (5 µg/mL), and AE was added at concentrations of 1.25, 2.5, and 5.0 µg/mL. After overnight incubation, the supernatants were harvested and frozen at −80 °C until cytokine determination. TNF-α and IL-6 in the supernatants were determined by enzyme immunoassays (ELISAs) according to the attached protocol of the kit.

#### 2.5.6. Induction of Cytokines in the HPVE-26 Cell Line

AE was added to a 24 h HPVE-26 cell culture at a final concentration of 10, 50, or 100 µg/mL. As a negative control, culture medium was applied. The cell cultures were stimulated with LPS (5 µg/mL). After overnight incubation at 37 °C under 5% CO_2_, the supernatants were harvested and frozen at −80 °C until cytokine determination. The TNF-α and IL-6 levels in the supernatants were determined by enzyme immunoassays (ELISAs) according to the attached protocol, and the level of NF-κB activation in the cells was determined by immunocytochemical staining.

#### 2.5.7. Cytokine Assay

Cytokine levels were determined in supernatants using ELISA kits for human TNF-α and IL-6 according to the manufacturer’s instructions. The optical density was measured at λ = 450 nm using a Multiskan RC spectrophotometric reader (Thermo Labsystems, Waltham, MA, USA).

#### 2.5.8. Immunocytochemical Staining for NF-κB

A well-established immunocytochemical method to assess the activation of the NF-κB system in PBMCs and HPVE-26 cells was applied. Cells were placed on poly-L-lysine-coated microscope slides using cytocentrifugation (Cytospin 4, Thermo Shandon, Cheshire, UK) (5 min at 500 rpm). Cells were fixed at RT using a 4% paraformaldehyde solution and air dried. After washing with distilled water, the endogenous peroxidase activity was blocked by incubating the slides in a 3% hydrogen peroxide solution in methanol for 5 min and washing with 10 mM PBS (pH = 7.5). Cytospin preparations were treated with universal blocking serum for 20 min at RT. Subsequently, cells were incubated at RT for another 60 min in a wet chamber with a polyclonal rabbit anti-NF-κB IgG antibody (p-65 subunit). After washing with PBS, the preparations were incubated with a biotinylated secondary anti-rabbit antibody at RT for 30 min. This was followed by washing with PBS and application of peroxidase-conjugated avidin in a wet chamber at RT for 30 min. After washing with PBS, chromogen fast DAB was used for 2–10 min. Subsequently, the preparations were counterstained in haematoxylin, washed for a final time with distilled water, and mounted on medium. Two independent blinded observers quantified the cytoplasmic or nuclear staining in cells (100 cells were counted on each slide). PBMCs and HPVE-26 cells expressing p-65 in the nucleus were labelled as NF-κB (+) cells. Activation of the NF-κB system in PBMCs and HPVE-26 cells was expressed as the percentage of NF-κB (+) cells among all quantified cells. NF-κB activation was evaluated using a Nikon type 120 microscope (Tokyo, Japan) with a video channel and appropriate computer software. The percentage of cells with stained nuclei reflected the degree of activation of the cells studied (100 cells were counted on each slide with 3 independent observations).

### 2.6. Ames Mutagenicity Test

**AE** mutagenicity studies were performed in an in vitro model via the Ames MPF 98/100 (Xenometrix, Endotell) microplate test using the *Salmonella typhimurium* strains TA98 and TA100 [36,37]. Briefly, 10 μL of each **AE** sample dissolved previously in DMSO in the concentration range of 15.6–500 µg/mL was mixed with 240 μL of bacterial suspension in exposure medium (with or without metabolic activation achieved by the Aroclor 1254-induced fraction of rat liver S9 (4% mix)) in 24-well plates, and the mixture was incubated (90 min, 37 °C) with shaking. Next, 2.8 mL of medium containing pH indicator was added to each well before shaking, and then 50 μL of each mixture was transferred to breeding medium and incubated (48 h, 37 °C) with shaking. Finally, wells with visible colonies or those where the colour of the mixture changed to violet as a result medium acidification due to CO_2_ production by bacteria during the metabolic process were counted; these wells were assumed to have the revertant present. Finally, wells with visible colonies of *S. typhimurium* or with a visible colour change to purple were counted and identified as having the his^+^ revertant present. The concentration of **AE** was considered mutagenic when it caused at least a threefold increase in the number of revertants compared to the negative control. To measure spontaneous reverse mutation, DMSO was used as a negative control, while several mutagens (2-nitrofluorenone (2-NF) for TA98, 4-nitrochinolin-*N*-oxide (4-NQ) for TA100, and 2-aminoanthracene (2-AA) for TA98S and TA100S) were used as positive controls.

### 2.7. Statistical Analysis

Statistical evaluation was carried out with Statistica software (13.1, TIBCO Software, Palo Alto, CA, USA). Data are expressed as the mean ± S.D. of at least 5 replicate experiments. Significant differences between the treated groups and control were determined by one-way ANOVA, Tukey HSD post hoc tests were used for examining statistical differences among more than two groups, and the Student’s *t*-test was used for two groups. A value of *p* < 0.05 was considered statistically significant.

## 3. Results and Discussion

### 3.1. Isolation and Structural Characterization of AE

**Figure 1:**
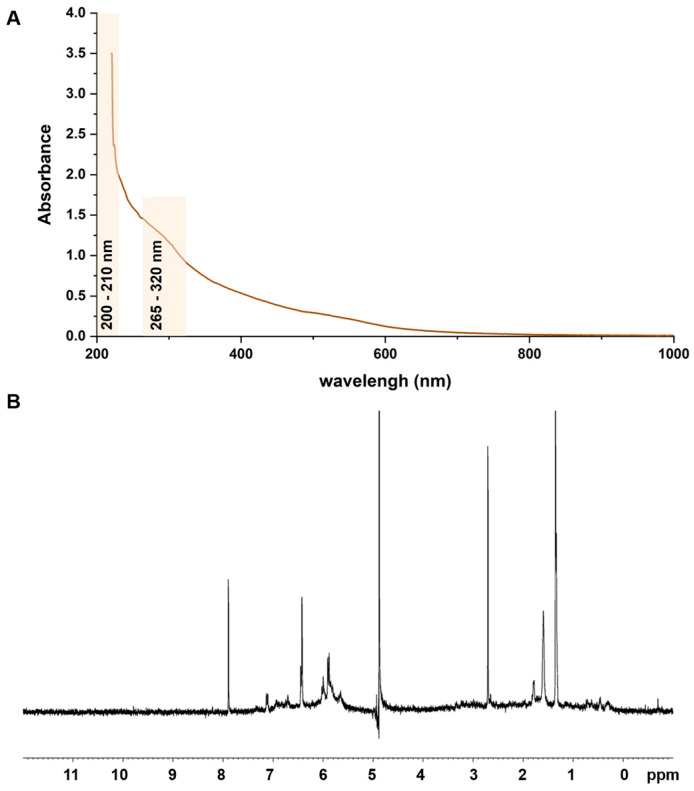
(**A**) UV–Vis and (**B**) ^1^H NMR spectra of AEag.

The complex biopolymer derived from *A. eupatoria* (**AE**) was obtained in the form of a water-soluble dark crimson precipitate. As previously reported, gel permeation chromatography (GPC) analysis revealed that the **AE** complex is macromolecular in nature (M_w_ = 55 × 10^3^ g/mol) and consists of a polysaccharide part and an aglycone (**AEag**), which is a lignin-like matrix [16]. Acid hydrolysis in 2 M TFA showed that **AEa**g represents approximately 48.4% of the whole product (*w/w*). From the former pyrolysis-methylation analysis of the aglycone component of **AE**, it was reported that the polyphenolic matrix is composed mostly of dimethoxyphenyl subunits [16]. The UV–Vis spectrum of **AEag** (Figure 1A) exhibited two discrete signals at λ~210 nm, which is characteristic of the hydroxyl groups of phenolic structures [38]. Furthermore, a wide band in the wavelength range of λ = 265–320 nm, typical for hydroxyl as well as carboxyl or carbonyl residues present in aromatic compounds [38], was noticed.

The ^1^H NMR spectrum of **AEag** (Figure 1B) revealed some protons associated with aromatic ring carbons, including those located near hydroxyl substituents in phenolic structures, such as the peaks in the range of δ ~ 7.2–6.7 ppm and slightly less intense signals with shifts of δ ~ 6.4 ppm and δ ~ 6.0–5.8 ppm, which in the combination confirmed the polyphenolic nature of the aglycone portion of **AE** [39,40,41]. However, the lack of peaks in the range of δ ~ 5.5–4.5 ppm, which are characteristic of protons associated with the anomeric carbon atoms of carbohydrates, indicated the absence of saccharide moieties in the analyzed sample. Likewise, the ^1^H NMR spectrum also showed a group of signals in the range of δ ~ 1.6–1.3 ppm that indicated the presence of aliphatic –CH_2_– fragments, a rather intense peak at approximately δ ~ 2.7 ppm that is characteristic of protons in –COCH_3_ groups, and a small multiplet at δ ~ 0.6–0.4 ppm from –CH_3_ protons. These structural elements are typically present in polyphenolic networks [39,40,41]. Summarizing the chemical analyses presented and our previous results, only substances with phenolic and carbohydrate characteristics were identified in **AE**, with the exception of a small amount of protein (~1%) [16].

### 3.2. Antioxidant Activity of AE

**Figure 2:**
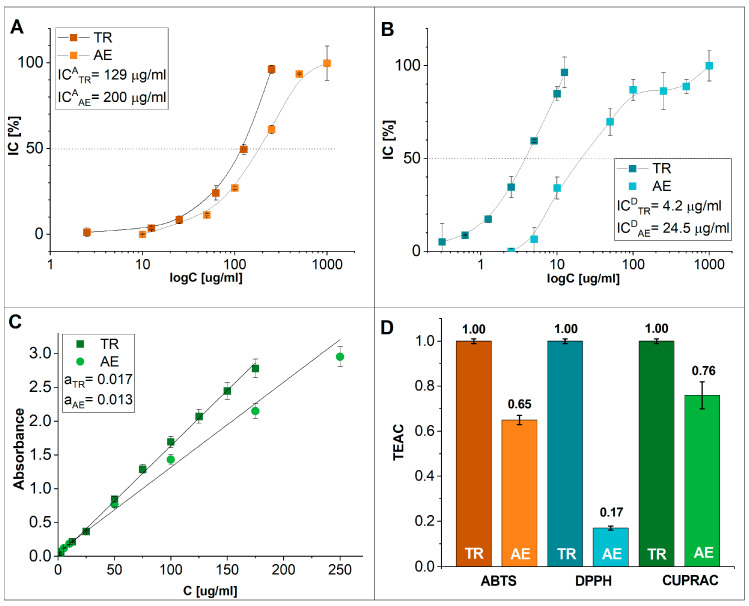
The ability of AE to scavenge (**A**) ABTS^●+^ and (**B**) DPPH^●^. (**C**) The reducing potency of AE measured by the CUPRAC assay. (**D**) Summary of the AE and TR IC_50_ values in which TR was used as a positive control.

The polyphenolic component of **AE** is a structurally diverse molecule and thus may contain both hydrophobic and hydrophilic elements. Therefore, to evaluate the total ability of **AE** to directly scavenge free radicals, tests typically used for the evaluation of the antioxidant activity of hydrophobic and hydrophilic substances were applied. The relative antioxidant activity of **AE** was calculated as TR equivalents and is expressed as TEAC (TEAC_TR_ = 1) (Figure 2D) and IC^A^_50_ and IC^D^_50_ values, which represent the concentration of the analyzed sample where of 50% ABTS^●+^ and DPPH^●^ were neutralized, respectively. The results were assessed using various polynomial equations and calculated from the standard curves prepared for TR and **AE**, which characterized the nonlinear dependence of IC on the test substance at a given concentration well. The ability of **AE** to neutralize the cationic form of ABTS radicals resulted in a TEAC^A^_AE50_ value of 0.65 and an IC^A^_AE50_ value of 200 µg/mL, whereas with TR, the activity was slightly lower (IC^A^_TR50_ = 129 µg/mL) (Figure 2A). **AE** neutralization of DPPH radicals (Figure 2B) gave a TEAC^D^ value = 0.17, which meant that IC^D^_AE50_ = 24.5 µg/mL, while the IC^D^_TR50_ value was 4.2 µg/mL. In the CUPRAC test, **AE** reduction of Cu^2+^ to Cu^+^ suggested even stronger free radical scavenging activity, as TEAC^C^_AE_ was only 25% lower than TEAC^C^_TR_ (Figure 2C). The results of antioxidant activity of AE, assessed in three different methods, are statistically significant (*p*-value < 0.05). This conclusion was confirmed by one-way ANOVA and post hoc Tukey HSD tests.

The high activity of **AE** to eliminate ABTS^●+^ and its ability to reduce copper suggest that this plant preparation may be an effective antioxidant that involves both HAT and SET; thus, **AE** can effectively lower the risk from harmful free radicals. The polysaccharide component of the plant product probably contributes to the reducing properties of **AE**, as it consists mainly of arabinogalactan bound to highly esterified rhamnogalacturonan [16]. Moreover, **AE** has good solubility in an aqueous medium and high free radical scavenging activity at neutral pH values close to physiological pH; thus, it may be assumed that **AE** could be effective in living systems. The evidently lower DPPH^●^ scavenging effect of **AE** may be due to several factors: (i) steric hindrance when the phenyl rings of DPPH^●^ prevent the effective donation of protons from the macromolecular phenolic **AE** hydroxyl groups to the unpaired electrons present on the nitrogen in the radical structure; and (ii) the reaction medium was an alcohol-water solution instead of pure alcohol due to the insolubility of **AE** in organic solvents. The results of the present work regarding the radical scavenging capacity (RSC) of **AE** are in agreement with previous studies on crude aqueous extracts of the herb *A. eupatoria*. The DPPH^●^ RSC value of the crude extracts was approximately 0.3, and that for ABTS^●+^ was at least two times higher [42,43,44]. However, **AE** neutralized free radicals more efficiently than the crude extracts of *A. eupatoria* due to the high purity of **AE**.

### 3.3. Determination of AE Toxicity

**Figure 3:**
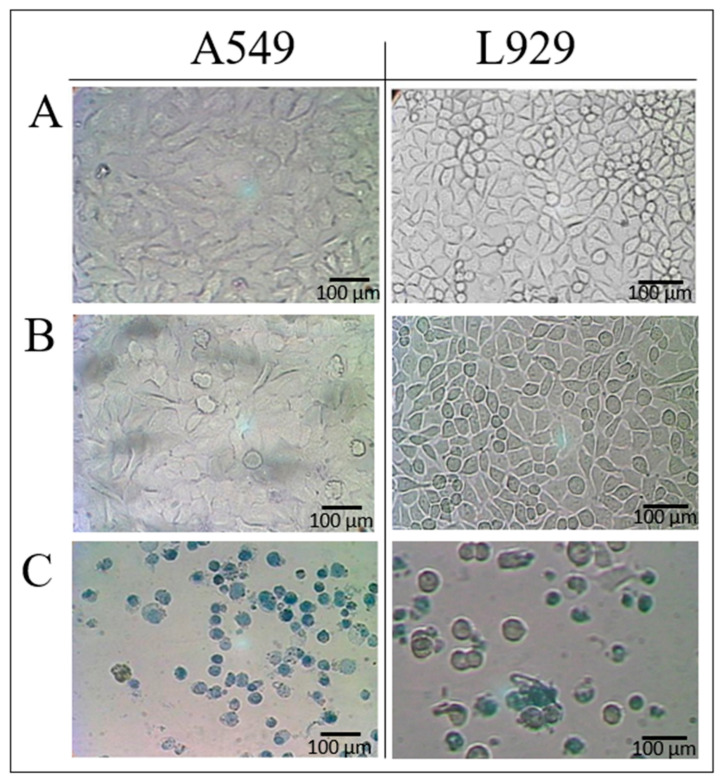
Photographs of A549 and L929 cell cultures after 72 h. (**A**) Live cells after incubation with culture media. (**B**) Live cells after incubation with AE (500 µg/mL) (no toxic effect). (**C**) Dead cells after incubation with AE (1000 μg/mL) (toxic effect).

The cytotoxicity of **AE** was determined by measuring the growth of human epithelial cell line A549 and mouse fibroblast cell line L929. The criteria for determining the toxicity effect were based on changes in cell morphology according to EN ISO10993-5:2009 [35]. The method involves the use of two types of cells—healthy and cancer cells. The metabolic activity of cell lines was evaluated using **AE** at concentrations ranging from 1.9–1000 µg/mL. The results showed that the *A. eupatoria* product was generally nontoxic up to 500 µg/mL (Table 1). A toxic effect from **AE** was observed only at a concentration of 1000 µg/mL. The lowest concentration of **AE** that was toxic to approximately 50% of the cells was expressed as TCCD_50_. The cell cultures incubated with **AE** were evaluated every 24 h using image analysis. The cells presented a normal shape and size, similar to those treated with culture medium (CM). A toxic effect of the plant product was found at a concentration of 1000 µg/mL, which was characterized by a change in cell morphology, the presence of granules in the cytoplasm, and subsequent cell death (Figure 3).

**Figure 4:**
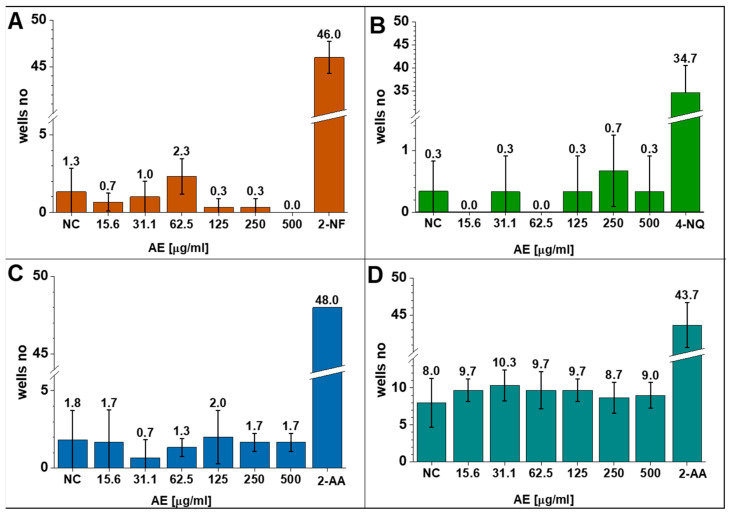
Numbers of positive wells from the Ames tests with *S. typhimurium* strains (**A**) TA98 and (**B**) TA100 without metabolic activation, and *S. typhimurium* strains (**C**) TA98S and (**D**) TA100S with metabolic activation. Values are expressed as the mean ± S.D.

Studies on the mutagenic and genotoxic potential of the *A. eupatoria* product were carried out by using the Ames test at doses that were verified as noncytotoxic to the A549 and L929 cell lines, that is, concentrations of **AE** in the range of 15.6–500 µg/mL. Experiments were performed with two bacterial strains to detect mutation points directly: *S. typhimurium* TA98 to read changes in the DNA frame shift and *S. typhimurium* TA100 to detect abnormal base pair substitutions in the DNA chain. Bacterial cells were not activated prior to the experiments. Therefore, measurements were made only for cells undergoing spontaneous expression (negative control), **AE**-induced cell expression, or cells with metabolic activation (TA98S and TA100S). The genome of histidine-dependent bacteria (his^+^) was deliberately deprived of the ability to synthesize histidine to reduce their survival rate in an amino acid-free culture medium. During the experiments, the strains were inoculated in medium containing trace amounts of histidine sufficient for only several cell divisions. The presence of bacterial cells under these conditions means that the genetic defect that caused the inability to produce bacterial histidine was repaired by reverse mutation. Furthermore, the microsomal S9 fraction was introduced into the culture medium to simulate the biochemical conditions of mammals. The Ames test without the S9 fraction allowed the detection of direct mutagens, while the test with the S9 fraction allowed the detection of indirect mutagens. Bacteria were also treated with known mutagens as positive controls, i.e., 2-NF, 4-NQ or 2-AA, to verify their genotoxic susceptibility. The results obtained with and without metabolic activation indicated that the **AE** product was not mutagenic at any of the doses tested in relation to the genomes of *S. typhimurium* TA98 and *S. typhimurium* TA100 (Figure 4). The *A. eupatoria* product at all concentrations tested did not induce reverse mutation, as the number of cells measured was similar or even lower than that in the negative control. Furthermore, all the tested doses of **AE** did not cause a concentration-dependent increase in the number of revertants. The differences between Ames tests results did not reach statistical significance (*p*-value < 0.05). These data allowed us to conclude that the plant product did not contain direct mutagens that cause reading phase shift and base pair substitution mutations in bacterial DNA, which can be detected by the test strains used. Pukalskiene et al. [42] and Santos et al. [45] reported that crude aqueous extracts of the herb *A. eupatoria* were nontoxic and nonmutagenic towards healthy cell lines, and even at rather high doses (C = 382 μg/mL), nearly 100% cell viability was observed. The in vitro cytotoxicity, mutagenicity, and genotoxicity studies proved that **AE** is potentially safe and could be recommended for experiments in an in vivo model.

### 3.4. Effect of AE on the Immune Response in the HPVE-26 Cell Line and in Whole Blood In Vitro

The aim of this research was to evaluate the direct effect of the **AE** product on the secretion of the cytokines TNF-α and IL-6 by human pulmonary vein endothelial cells (HPVE-26 cells) and whole blood culture in vitro, as well as to determine the nuclear activation of NF-κB in HPVE-26 cells and PBMCs. Proinflammatory cytokines are products of inflammatory reactions in the body, and the expression of these proteins is controlled by regulatory transcription factors, including NF-κB. Furthermore, NF-κB is known to be critical in the regulation of proinflammatory molecules during cellular responses, particularly the expression of cytokines, i.e., interleukin-1β (IL-1β), TNF-α, interleukin-8 (IL-8), IL-6, and interferon-β (IFN-β). In most eukaryotic cells, the NF-κB signal transduction pathway has been found to be responsible for the regulation of numerous biochemical processes and immunological responses. Activation of NF-κB is considered an important initial event in the inflammatory response to a variety of stimuli, including infective agents, toxins, cytokines, growth factors, oxidative stress, and changes in physical conditions [46,47]. After cell stimulation, information from the external environment is transduced to the interior of the cell, and gene expression is turned on. The activation of NF-κB is a multistep process that consists of phosphorylation, ubiquitinoylation, and the degradation of inhibitor subunits connected to the functional dimer of NF-κB proteins [48]. NF-κB proteins are present in the cytoplasm in an inactive form combined with their inhibitor IκB. When phosphorylated IκB dissociates from the NF-κB-IκB complex, it results in the translocation of NF-κB from the cytoplasm to the nucleus. IκB molecules undergo degradation under the influence of an appropriate kinase, enabling the translocation of NF-κB from the cytoplasm to the nucleus, its binding with an appropriate gene sequence, the activation of transcription, and the production of proinflammatory cytokines [46]. The numerous genes involved in the inflammatory response are regulated by NF-κB activity (Gilmore Lab Publications » NF-KB Transcription Factors|Boston University, n.d.) [49]. NF-κB is highly activated at sites of inflammation in various diseases, such as multiple sclerosis, inflammatory bowel diseases, psoriasis, and asthma.

**Figure 5:**
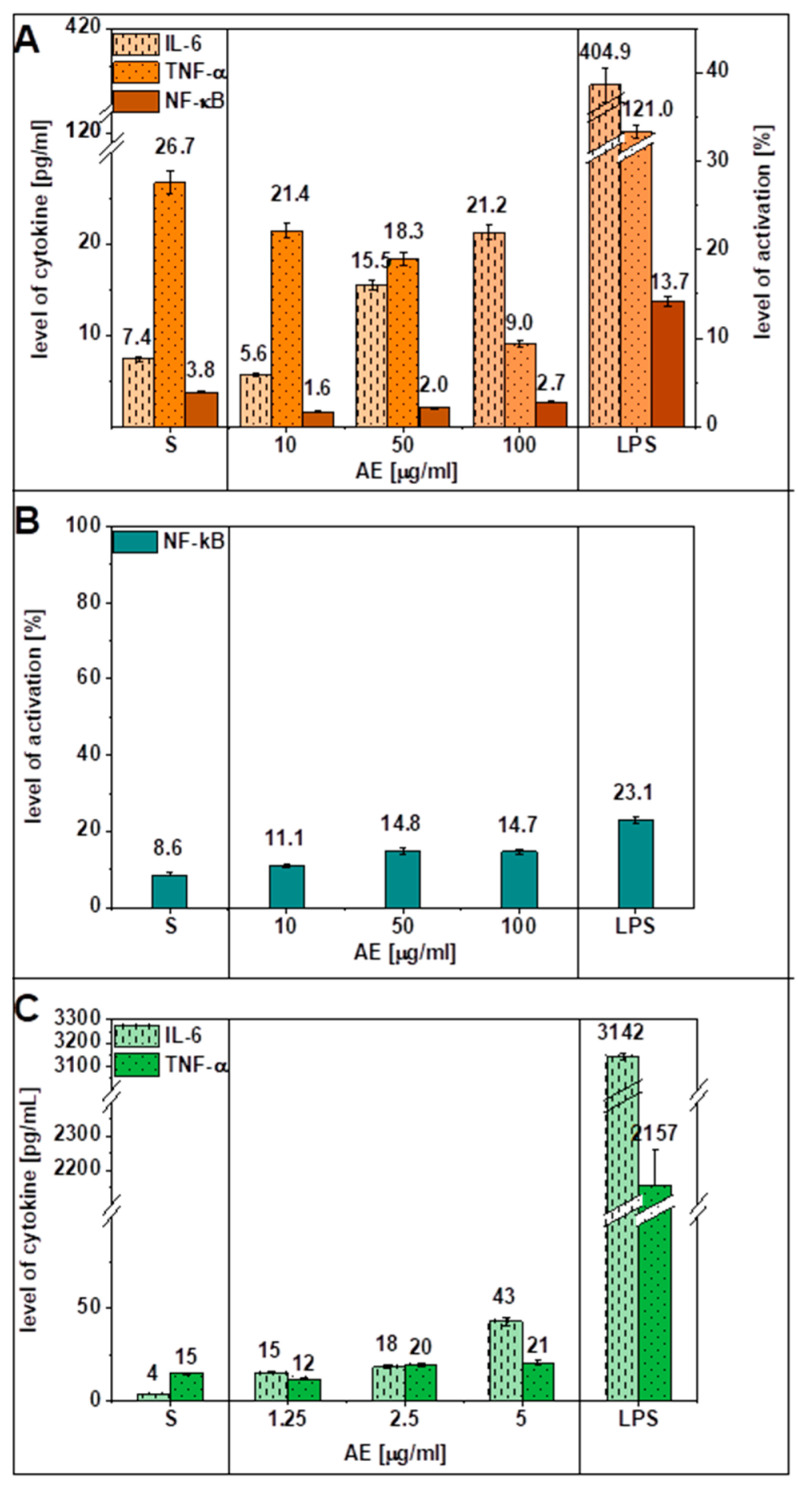
The effects of AE and LPS (5 μg/mL) on the production of the cytokines TNF-α and IL-6 (**A**) by human pulmonary vein endothelial cells (HPVE-26 cells) and (**C**) in human whole blood culture in vitro. The effects of AE and LPS on the activation of (**A**) NF-κB in HPVE-26 cells and (**B**) PBMCs. S stands for spontaneous secretion of cytokines (**A**,**C**) or spontaneous activation of NF-κB (**B**). Values are expressed as the mean ± S.D.

The results here showed that **AE** did not stimulate human HPVE-26 cells to produce higher amounts of TNF-α and IL-6, while LPS (endotoxin) caused the release of these cytokines in large amounts. LPS stimulation of human HPVE-26 cells increased the concentration of TNF-α from 26.7 pg/mL to 121.0 pg/mL and the concentration of IL-6 from 7.39 to 404.91 pg/mL (Figure 5A). However, the correlation between levels of activated NF-κB and the amounts of TNF-α secreted by human HPVE-26 cells, in the presence of a series of concentrations of AE, were statistically significant. Similarly, in whole blood culture in vitro, AE did not induce an increase in the amounts of both analyzed cytokines, but LPS did cause an increase in the production of TNF-α from 14.7 to 2156.9 pg/mL. The level of IL-6 in whole blood culture also increased from 3.6 pg/mL to 3142.3 pg/mL, after stimulation by LPS (Figure 5C). The results of analysis of TNF-α amounts and proinflammatory cytokines concentrations obtained in human whole blood samples, in the presence of AE, regardless of its tested concentration, did not reach statistical significance between the examined groups (F-value = 0.593321), what was confirmed in post hoc Tukey HSD test.

**Figure 6:**
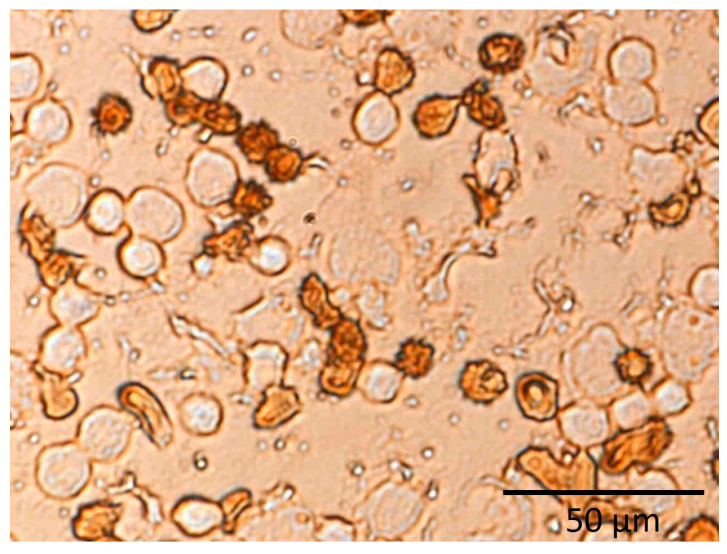
In vitro human peripheral blood mononuclear cells showing the active form of NF-κB (dark brown) and the inactive form of NF-κB (light brown).

Furthermore, no significant differences in NF-κB expression were observed in HPVE-26 cells or in PBMCs from healthy individuals after induction by various concentrations of **AE** compared to the control group (Figure 5A,B). In PBMCs obtained from healthy individuals and HPVE-26 cells, stimulation with LPS (5 µg/mL) increased the activation of NF-κB, as evidenced by translocation of p-65 to the nucleus (Figure 6). The percentages of NF-κB (+) in PBMCs or HPVE-26 cells after control stimulation without LPS vs. stimulation with LPS increased from 8.6% to 23.1% in PBMCs and from 3.8% to 13.7% in HPVE-26 cells. After LPS stimulation of blood cells from healthy donors and HPVE-26 cells, NF-κB activation was detected at levels found in various diseases. The authors of previous studies demonstrated that polyphenols activate heterodimers of the NF-E2-related factors/antioxidant responsive element pathway, which, in turn, can modify the activity of NF-κB [50,51]. This finding suggested that **AE** was able to reduce the expression of proinflammatory cytokine mRNAs, such as TNF-α and IL-1β, in blood cells exposed to LPS. Hougee et al. [52] found that flavonoids and various glycosides, such as luteolin, apigenin, and other compounds isolated from the herb *A. eupatoria*, can reduce NF-κB transcriptional activity in LPS-stimulated primary monocytes [53] and macrophages [54].

Various studies have demonstrated the anti-inflammatory potential of *Agrimonia* species [55,56]. Although the mechanisms of the anti-inflammatory effects of plant extracts have not yet been fully elucidated, recent data have shown that **AE** extracts are capable of reducing the activity of NF-κB via suppression of its nuclear translocation [57].

In summary, the particularly notable finding of this study is that **AE** did not cause a significant inflammatory response. Furthermore, in the HPVE-26 cell line, the *A. eupatoria* product slightly reduced the level of proinflammatory cytokines and the activity of the NF-κB below the physiological level. Experiments with PBMCs revealed that **AE** did not induce activation of the NF-κB above the level of spontaneous activation. Taking these results as promising, one may suggest that **AE** does not stimulate cells to produce inflammatory mediators and at certain doses may even protect cells from inflammation. In addition, Santos et al. [45] confirmed an anti-inflammatory effect of crude aqueous extracts of *A. eupatoria* in mice in vivo for inflammation-related pathologies.

## 4. Conclusions

The presented research is a continuation of the structural and biological studies of the complex biopolymer isolated from the flowering parts of *A. eupatoria* L. (**AE**). Spectroscopic studies (UV–Vis and ^1^H NMR) of the **AE** aglycone component confirmed its polyphenolic characteristics. Regarding its biological properties, **AE** significantly eliminated ABTS^·+^ and DPPH^·^ species and acted as an effective copper reducing agent in the CUPRAC test, ultimately proving its powerful antioxidant properties. Furthermore, **AE** was nontoxic to A549 human lung adenocarcinoma cells and L929 mouse fibroblasts. Moreover, **AE** was neither genotoxic nor mutagenic to *S. typhimurium* strains TA98 and TA100. Additionally, the presented results showed that **AE** did not induce the release of proinflammatory cytokines (IL-6 and TNF-α) by endothelial cells of the human pulmonary vein (HPV26) or human PBMCs, which correlated with low the activation of NF-κB in these cells.

The protective ability of **AE** to prevent the negative influence of free radicals due to its polyphenol component, together with the nontoxicity and anticoagulant effect from its pectin-like component [16], which increase **AE** solubility in the physiological environment, display its potential to perform a highly desirable function in the bloodstream, such as preventing numerous unwanted events in the circulatory system. Free radicals are a common cause of blood vessel inflammation and can lead to uncontrolled thrombotic processes. Therefore, the *A. eupatoria* product offers some prospects for the future, such as prophylactic and supporting therapies for inflammatory conditions of various etymologies, including societal diseases. **AE** may protect the human body from oxidative stress and consequently also reduce the risk of the formation of secondary reactive oxygen and nitrogen species, both of which excessively induce various cellular responses that ultimately lead to cell necrosis and even apoptosis. Thus, **AE** may be considered a functional ingredient for pharmaceutical, food, and cosmetic applications.

## Figures and Tables

**Table 1:** The toxic effect of AE in L929 and A549 cells in vitro. Culture medium (CM) was used as a negative control.

Cell Line	Sample	Concentration [μg/mL]									
		1000	500	250	125	62.5	31.2	15.6	7.8	3.9	1.9
L929	**AE**	T	N	N	N	N	N	N	N	N	N
	CM	N	N	N	N	N	N	N	N	N	N
A549	**AE**	T	N	N	N	N	N	N	N	N	N
	CM	N	N	N	N	N	N	N	N	N	N

T: toxic effect; N: no toxic effect.

## Data Availability

Data available on request due to project restrictions.
